# Efficient Generation of Functional Dopaminergic Neurons from Human Induced Pluripotent Stem Cells Under Defined Conditions

**DOI:** 10.1002/stem.499

**Published:** 2010-08-16

**Authors:** Andrzej Swistowski, Jun Peng, Qiuyue Liu, Prashant Mali, Mahendra S Rao, Linzhao Cheng, Xianmin Zeng

**Affiliations:** a Laboratory for Stem Cells & Aging, Buck Institute for Age ResearchNovato, California, USA; b Johns Hopkins Institute for Cell Engineering, Johns Hopkins University School of MedicineBaltimore, Maryland, USA; c Department of Biomedical Engineering, Johns Hopkins University School of MedicineBaltimore, Maryland, USA; d Life Technologies, CarlsbadCalifornia, USA; e Department of Medicine, Johns Hopkins University School of MedicineBaltimore, Maryland, USA

**Keywords:** Human iPSC, Human ESC, Dopaminergic neuron, Neural stem cell

## Abstract

Human induced pluripotent stem cells (iPSCs) reprogrammed from somatic cells represent a promising unlimited cell source for generating patient-specific cells for biomedical research and personalized medicine. As a first step, critical to clinical applications, we attempted to develop defined culture conditions to expand and differentiate human iPSCs into functional progeny such as dopaminergic neurons for treating or modeling Parkinson's disease (PD). We used a completely defined (xeno-free) system that we previously developed for efficient generation of authentic dopaminergic neurons from human embryonic stem cells (hESCs), and applied it to iPSCs. First, we adapted two human iPSC lines derived from different somatic cell types for the defined expansion medium and showed that the iPSCs grew similarly as hESCs in the same medium regarding pluripotency and genomic stability. Second, by using these two independent adapted iPSC lines, we showed that the process of differentiation into committed neural stem cells (NSCs) and subsequently into dopaminergic neurons was also similar to hESCs. Importantly, iPSC-derived dopaminergic neurons were functional as they survived and improved behavioral deficits in 6-hydroxydopamine-leasioned rats after transplantation. In addition, iPSC-derived NSCs and neurons could be efficiently transduced by a baculoviral vector delivering episomal DNA for future gene function study and disease modeling using iPSCs. We also performed genome-wide microarray comparisons between iPSCs and hESCs, and we derived NSC and dopaminergic neurons. Our data revealed overall similarity and visible differences at a molecular level. Efficient generation of functional dopaminergic neurons under defined conditions will facilitate research and applications using PD patient-specific iPSCs. Stem Cells 2010;28:1893–1904

## INTRODUCTION

Parkinson's disease (PD) is a common and ultimately incapacitating disease with no cure. The main pathological hallmark of PD is a progressive loss of substantia nigra dopaminergic neurons in the midbrain. The availability of human embryonic stem cells (hESCs) and methods for in vitro differentiation of dopaminergic neurons from hESCs [1–4], animal models of PD, and previous encouraging results from fetal transplants have raised the possibility that one may be able to manufacture human dopaminergic neurons from hESCs in unlimited quantities in vitro. Indeed, we and others have provided evidence for a scalable good manufacture practice (GMP) process that will allow one to obtain dopaminergic neurons from multiple hESC lines [5].

The recently acquired ability to reprogram human adult somatic cells to induced pluripotent stem cells (iPSCs) in culture [6] has raised the possibility that not only could we provide allogenic dopaminergic neurons but also now provide an unlimited source of personalized cells for replacement therapy. Human iPSCs thus may allow us to bypass the immunorejection issue faced with allogenic cell transplants and may also solve bioethical concerns surrounding hESCs. As PD may be either hereditary or acquired, iPSC lines derived from such patients could also serve as a unique tool for drug discovery or therapeutic cell replacement or gene delivery applications. Several key issues, however, need to be addressed before iPSC-based therapy can become prevalent. This requires extending the development of defined cell culture systems developed for hESC-derived dopaminergic neurons, confirming that iPSC lines are overall similar to hESCs, and developing zero footprint iPSC induction technology and efficient gene delivery as well as retargeting methodologies.

Although there are few side by side comparisons directed at revealing subtle differences between ESCs and iPSCs, published results so far, largely confirm that irrespective of the path to pluripotency, the cells behave virtually similarly to each other. Nevertheless, some differences have been observed. The frequency of karyotypic abnormalities in iPSCs seems to be higher than in ESCs. Anecdotal evidence also suggests that teratomas from iPSCs appear less complex and more cystic. The frequency and extent of chimerism with mouse iPSCs is more limited and there appear biases depending on the cell of origin of the pluripotent population. More recently, differences between iPSCs and ESCs in gene expression have been reported [7], however, whether these differences are significant and wider than normal allelic differences remains to be seen.

Ultimately, it is important to address fundamental issues such as whether human iPSCs can efficiently differentiate into therapeutically relevant cells like dopaminergic neurons as hESCs can, and whether such cells are functional both in vitro and in vivo. Although neurons expressing tyrosine hydroxylase (TH) have been generated from human iPSCs [8,9] and functional dopaminergic neurons have been derived from mouse iPSCs [10], it remains unknown whether authentic substantia nigra (A9) dopaminergic neurons can be efficiently generated from human iPSCs, and whether such cells will function in vivo for potential cell therapy. Thus, there is a lack of robust culture systems for efficient generation of functional A9 dopaminergic neurons from human iPSCs.

In this study, we have validated a scalable protocol for efficient generation of A9 dopaminergic neurons from multiple iPSC lines using a completely defined xeno-free system that we have developed for hESC differentiation [5]. Using this procedure, we showed that neural stem cells (NSCs) derived from two human iPSC lines adapted to defined media were able to differentiate into functional dopaminergic neurons similar to hESCs in terms of time course, neural patterning, and efficiency of generation of dopaminergic neurons. Side by side comparison of iPSCs and hESCs as well as of iPSC- and hESC-derived NSCs and dopaminergic neurons revealed that iPSCs were overall similar to hESCs in gene expression profiles. In addition, we showed that iPSC-derived dopaminergic neurons could improve symptoms of PD in a preclinical rodent model, and be genetically modified efficiently. Our approach will facilitate subsequent adaptation of protocols to GMP standards which is a prerequisite for progression toward clinical trials.

## MATERIALS AND METHODS

### Cell Culture

The iPSC lines MR31 (derived from IMR90 fetal lung fibroblasts) and MMW2 (derived from adult mesenchymal stem cells from bone marrow) were derived as described [11]. At passages 10 and 15, cells were adapted to a completely humanized-base medium consisting of StemPro hESC medium with 20% human Knockout Serum Replacement (hKSR), 1× StemPro protein cocktail and 8 ng/ml of basic fibroblast growth factor (FGF2) on culture dishes coated with CellStart, a xeno-free defined substrate (all from Invitrogen, Carlsbad, CA http://www.invitrogen.com) [5]. Cells were split every 4–6 days using a cell scraper and karyotyped every 10 passages. hESC culture was obtained as described previously [5].

### Derivation of NSCs from iPSCs in Defined Media

iPSC colonies (>10 passages in defined medium with a normal karyotype) were harvested using a scraper and cultured in suspension as embryoid bodies (EBs) for 8 days in StemPro defined medium minus FGF2. EBs were then cultured for additional 2–3 days in suspension in neural induction medium containing Dulbecco's Modified Eagle Medium (DMEM/F12) with Glutamax, NEAA, N2, and FGF2 (20 ng/ml) prior to attachment on cell culture plates coated with CellStart. Neural rosettes formed 2–3 days after adherent culture were isolated manually using stretched glass Pasteur pipette and placed in fresh culture dishes. Then, the rosettes were dissociated into single cells using accutase and replated onto culture dishes to obtain a homogeneous population of NSCs. The NSCs population was expanded in Neurobasal media containing NEAA, 2 mM glutamine, B27, and 20 ng/ml FGF2.

To confirm that NSCs can differentiate into astrocytes, NSCs were cultured in DEMEM/F12 medium supplemented with 1× NEAA, l-glutamine (2 mM), 1× N2, and 1× B27 for 2 weeks and processed for immunostaining. To initiate oligodendrocyte differentiation, NSCs were cultured in medium containing DMEM/F12, 1× NEAA, l-glutamine (2 mM), 1× N2, 1× B27, Shh (200 ng/ml, R&D Systems), and retinoic acid (RA; 2 μM) for 10 days, and then with NT3 (30 ng/ml, R&D Systems) and platelet-derived growth factor alpha (PDGFα) (10 ng/ml, R&D Systems) but without Shh and RA for an additional 2 weeks.

### Dopaminergic Differentiation of NSCs

Dopaminergic differentiation in defined media was initiated by culturing NSCs for 10 days in neurobasal medium supplemented with NEAA, l-glutamine (2 mM), B27, Shh (200 ng/ml), and FGF8 (100 ng/ml). Shh and FGF8 were then withdrawn and replaced with Brain-derived neurotrophic factor (BDNF) and Glial cell-derived neurotrophic factor (GDNF) (20 ng/ml of each), Transforming growth factor beta-3 (TGFβ3) (1 μM), ascorbic acid (200 μM), and cAMP (1 mM) for 3 weeks (∼30 days after the NSC stage).

### Immunocytochemistry

Immunocytochemistry and staining procedures were as described previously [12]. Briefly, hESCs at different stages of dopaminergic differentiation were fixed with 2% paraformaldehyde for half an hour. Fixed cells were blocked in blocking buffer (10% goat serum, 1% BSA, 0.1% Triton X-100) for 1 hour followed by incubation with the primary antibody at 4°C overnight in 8% goat serum, 1% BSA, 0.1% Triton X-100. Appropriately coupled secondary antibodies (Molecular Probes, Carlsbad, CA http://www.invitrogen.com/site/us/en/home/brands/Molecular-Probes.html) were used for single and double labeling. All secondary antibodies were tested for cross reactivity and nonspecific immunoreactivity. The following primary antibodies were used: Oct4 (ab19857 AbCam, Cambridge, MA http://www.abcam.com) 1:1,000; β-Tubulin isotype III clone SDL.3D10 (T8660 Sigma, St. Louis, MO http://www.sigmaaldrich.com/united-states.html) 1:500; GalC (MAB342 Millipore, Billerica, MA http://www.millipore.com) 1:50; Glial fibrillary acidic protein (GFAP) (M0761 Chemicon) 1:50; Nestin (611658 BD Transduction laboratories) 1:500; Sox1 (AB5768 Chemicon Billerica, MA http://www.millipore.com) 1:800, TH (Pel-freez, Rogers, AR http://www.pelfreez-bio.com P40101) 1:500, TH clone TH-16 (T2928 Sigma) 1:1,000; Girk2 (Alamone) 1:250; Musashi (eBioscience, San Diego, CA http://www.ebioscience.com) 1:50, and as secondary antibodies: Alexa Fluor 488 Goat Anti-Mouse, Alexa Fluor 594 Goat Anti-Mouse, Alexa Fluor 488 Goat Anti-Rabbit, Alexa Fluor 594 Goat Anti-Rabbit. Hoechst 33342 (Molecular Probes H3570) 1:1,000 were used for nuclei identification. Images were captured on a Nikon fluorescence microscope.

The quantification of TH^+^ cells in culture was performed by analyzing fluorescent images using Photoshop on a minimum of 5,000 cells of at least 10 randomly chosen fields derived from three or more independent experiments. The number of Hoechst-labeled nuclei on each image was referred as total cell number (100%).

### Gene Expression by Microarray and Polymerase Chain Reaction Analysis

RNAs isolated from ESC/iPSC, NSCs, and dopaminergic populations were hybridized to Illumina Human HT-12 BeadChip (Illumina, Inc., San Diego, CA; performed by Microarray core facility at the Burnham Institute for Medical Research). Array data processing and analysis was performed using Illumina BeadStudio software. The Illumina array data were normalized by the background method. The maximum expression value for probe set of one gene was chosen as the expression value of this gene. Using the processed data, we conducted Global array clustering of genes across all the hESC/iPSC, NSC, and dopaminergic samples, using the complete linkage method and measuring the Euclidean distance. The result was presented by a dendrogram. Differentially expressed gene was defined if the gene showed twofold expression change between any two samples. The data of differentially expressed genes were transformed to log2 signal values for each gene across all samples. Unsupervised two-way hierarchical clustering of differentially expressed genes was analyzed with The Institute for Genomic Research Multiexperiments Viewer (MEV) v4.5.1 [13], which used complete linkage and Euclidean distance metric to generate the hierarchical tree. High expressions relative to mean are colored red, whereas low expressions are colored green. Black represents no significant change in expression level between mean and sample. All cell line correlations were a measure of Pearson's rho implemented in Statistical Analysis System (SAS).

cDNA was synthesized by using a reverse transcription kit SuperScript III First-Strand Synthesis System for Real Time Polymerase chain reaction (RT-PCR) (Invitrogen) according to the manufacturer's recommendations. Real-time polymerase chain reaction (PCR) was used to quantify the levels of mRNA expression of 10 genes in day 32 dopaminergic populations. PCR reactions were carried out by ABI 900HT instrument according to the manufacturer's instructions. Primer sequences were previously described [5].

### Baculovirus Preparation and Transduction

Baculoviral vector carrying a GFP driven by the Cytomegalovirus (CMV) promoter was obtained from Life Technologies and viral particles were prepared accordingly to manufacture's instruction. For transduction, NSCs were infected with bacculoviral particles at ratio of 500–1,000 Multiplicity of Infection (MOI) for 30 minutes at room temperature on a rocker. Transduced cells were plated on culture dishes without virus removal and cultured over night at 37°C. Viral particles were removed by a medium change next day. Neurons in adherent culture were transduced at the ratio as above and were incubated with the viral particles over night at 37°C. Medium was changed the next day.

### Transplantation into the 6-Hydroxydopamine Rats, Behavioral and Histological Analyses

Fourteen rats were anesthetized and 20 μg of 6-hydroxydopamine (6-OHDA; Sigma-Aldrich) were stereotaxically injected at a concentration of 4 μg/μl (in 0.2 mg/ml ascorbate in saline) at one site in the MFB (stereotaxic coordinates: anteroposterior, −4.4 mm; mediolateral, −1.2 mm; dorsoventral, −7.8 mm) using a Hamilton syringe. The toxin was injected at a rate of 1 μl/minute. To ensure complete lesion of the nigrostriatal DA pathway, the animals were screened by amphetamine-induced rotation at a dose of 2.5 mg/kg. Only animals [10] that exhibited a mean ipsilateral rotation score of seven or more complete body turns per minute were included in the study.

For rational behavior analysis, the animals were given 2.5 mg/kg d-amphetamine intraperitoneally, and their rotational behavior was monitored over a 90-minute period using the rotameter system (TSE) (Bad Homburg, Germany). Rotation toward the lesion (ipsilateral) was scored as positive and net rotational asymmetry score were expressed as full body turns per minute.

For histological analysis, brains were removed and immersion-fixed overnight at room temperature. Brains were then dehydrated in graded ethanol, cleared in xylene, and paraffin-embedded [14]. Seven micrometer-thick serial coronal sections were cut and mounted on glass slides, which were dried overnight at 42°C. Sections were deparaffinized, rehydrated through a graded series of ethanol, and washed in water. For immunostaining [15], sections were incubated with blocking solution (2% horse serum, 1% bovine serum albumin, and 0.1% Triton X-100 in phosphate-buffered saline, pH 7.5) and then with primary antibodies at 4°C overnight followed by secondary antibodies in blocking solution at room temperature for 2 hours. The primary antibodies used were rabbit anti-TH antibody (1:500; Pel-freez, Rogers, AR http://www.pelfreez-bio.com, P40101) and mouse anti-human nuclear antibody (1:300; Chemicon Billerica, MA http://www.millipore.com, MAB 1281). The secondary antibodies were rhodamine-conjugated rat-absorbed donkey anti-rabbit IgG (Jackson ImmunoResearch Laboratories, West Grove, PA http://www.jacksonimmuno.com; 1:200) and fluorescein isothiocyanate-conjugated pig anti-mouse IgG (Vector Laboratories Burlingame, CA http://www.vectorlabs.com; 1:200). Nuclei were counterstained with 4′,6-diamidino-2-phenylindole using proLong Gold antifade reagent (Invitrogen, Carlsbad, CA http://www.invitrogen.com). To quantitatively analyze double-labeled neurons in the striatum, fluorescence signals were detected with an LSM 510 NLO Confocal Scanning System mounted on an Axiovert 200 inverted microscope (Carl Zeiss Ltd Maple Grove, MN http://www.zeiss.com) equipped with a two-photon Chameleon laser (Coherent Inc Santa Clara, CA http://www.coherent.com). Three-color images were scanned using Argon and 543 HeNe lasers. IMARIS (Bitplane AG, Zurich Switzerland, http://www.bitplane.com) imaging software was used for three-dimensional image reconstruction. Images were acquired using LSM 510 Imaging Software (Carl Zeiss Ltd Maple Grove, MN http://www.zeiss.com) as described previously [15]. The specificity of each label was first verified using single-channel scans that were then merged into multiple-channel views. Neurons were considered double-labeled if colabeling with relevant morphology was seen throughout the extent of the nucleus for nuclear markers or if a cytoplasmic marker surrounds a nuclear marker when viewed in *x-y* cross section as well as in *x-z* and *y-z* cross-sections produced by orthogonal reconstructions from *z*-stacks taken at ×400 magnification. Human antigen single-labeled neurons and neurons double-labeled for TH and human antigen were recorded in every seventh section per animal (*n* = 3). The area of each transplanted region was simultaneously determined for each of the scored sections.

## RESULTS

### Adaption of Multiple iPSC Lines to Defined Media and Generation of NSCs from Adapted iPSCs

We have previously shown that hESCs can be maintained in a xeno-free environment and induced to differentiate into NSCs and subsequently to authentic dopaminergic neurons using animal origin-free components by a four-step scalable protocol [5]. To test whether iPSCs could be adapted to defined medium culture while retained genetic integrity and maintained the ability to generate multipotent NSCs after prolonged culture, we cultured and differentiated two human iPSC lines MMW2 and MR31 using identical components as for hESCs. The MMW2 line was derived from adult mesenchymal stem cells by the standard four retroviral vectors expressing the four factors, Oct4, Sox2, Klf4, and c-Myc [11]. The MR31 line was reprogrammed by only three factors (omitting c-myc) from human fetal fibroblasts [16]. Both lines were shown to be pluripotent and karyotypically normal [17].

**Figure 1:**
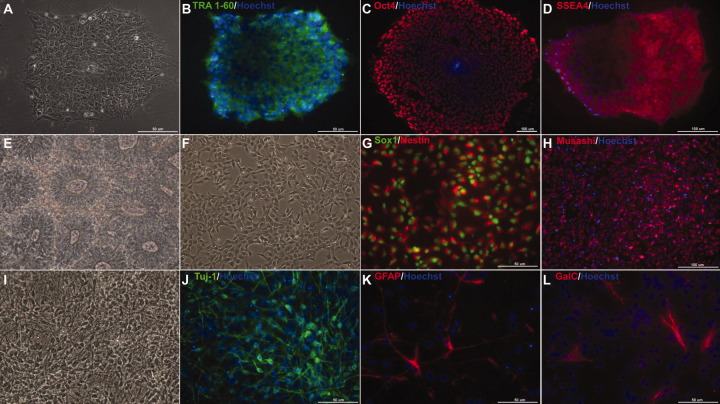
Generation of neural stem cells (NSCs) from induced pluripotent stem cell (iPSC) lines adapted to defined medium. iPSC line MR31 at passage 15 was adapted to a chemically defined medium StemPro. **(A–D):** Morphology **(A)** and expression of the pluripotent markers Tra 1-60 **(B)**, Oct4 **(C)**, and SSEA4 **(D)** in iPSCs that were cultured in StemPro for 10 passages. **(E–H):** Generation of NSCs in defined conditions. Neural tube-like rosette structures **(E)** were formed in the center of the iPSC colonies after 12 days of differentiation. A monolayer of homogeneous NSCs **(F)** coexpressed Sox1 and nestin **(G)**, and Musashi **(H)**. **(I–L):** iPSC-deriver NSCs retained the capacity to differentiate into neurons **(I–J)**, astrocytes **(K)**, and oligodendrocytes **(L)**. Abbreviation: GFAP, Glial fibrillary acidic protein.

To adapt iPSCs to a defined medium culture, early MR31 and MMW2 lines (at passages 10–15) were continuously cultured in StemPro medium (a chemically defined medium) on a defined substrate (CellStart) for over 10 passages. As seen in Figure 1A–1D, like hESCs, both iPSC lines grown in defined medium expressed pluripotentcy markers such as Oct4, TRA-1-60 and SSEA4, and maintained a normal karyotype in prolonged culture. No differences were observed in the course of adaption regarding morphological characteristics between hESCs and iPSCs. As the results are similar for both iPSC lines, staining images of only one of the lines (MR31) are shown here.

To generate NSCs, feeder-free defined medium cultured iPSC colonies were detached and cultured in suspension as EBs in a defined medium followed by adhered culture. Both iPSC lines formed neural tube-like rosette structures morphologically undistinguishable from those differentiated from hESCs (Fig. 1E). These rosette-derived cells uniformly expressed NSC markers nestin, Sox1, and musashi (Fig. 1G–1H), but not differentiated neuronal (e.g., β-III tubulin) or glial markers (GFAP or O4; data not shown). We did not observe significant differences between the two iPSC lines and the hESCs regarding the efficiency of generation of neural rosettes and NSCs. Furthermore, NSCs that were expanded in defined medium for over 10 passages maintained a normal karyotype and the expression of NSC markers Sox1 nestin and musashi. They also retained the ability to differentiate into neurons, astrocytes, and oligodendrocytes (Fig. 1I–1L). This result indicates that similar to hESCs, iPSCs adapted to defined medium differentiate into neural cells under defined conditions.

### iPSC-Derived NSCs Differentiated Efficiently into Dopaminergic Neurons in Defined Conditions

One of the major goals of using iPSCs for personalized medicine and disease modeling is to generate therapeutically target cells such as functional neurons of specialized neurotransmitter phenotype. We have previously shown that several hESC lines differentiate efficiently and similarly into authentic dopaminergic neurons using our xeno-free defined protocol [5], and therefore, we wish to address whether iPSC-derived NSCs can differentiate into functional dopaminergic neurons using an identical protocol.

**Figure 2:**
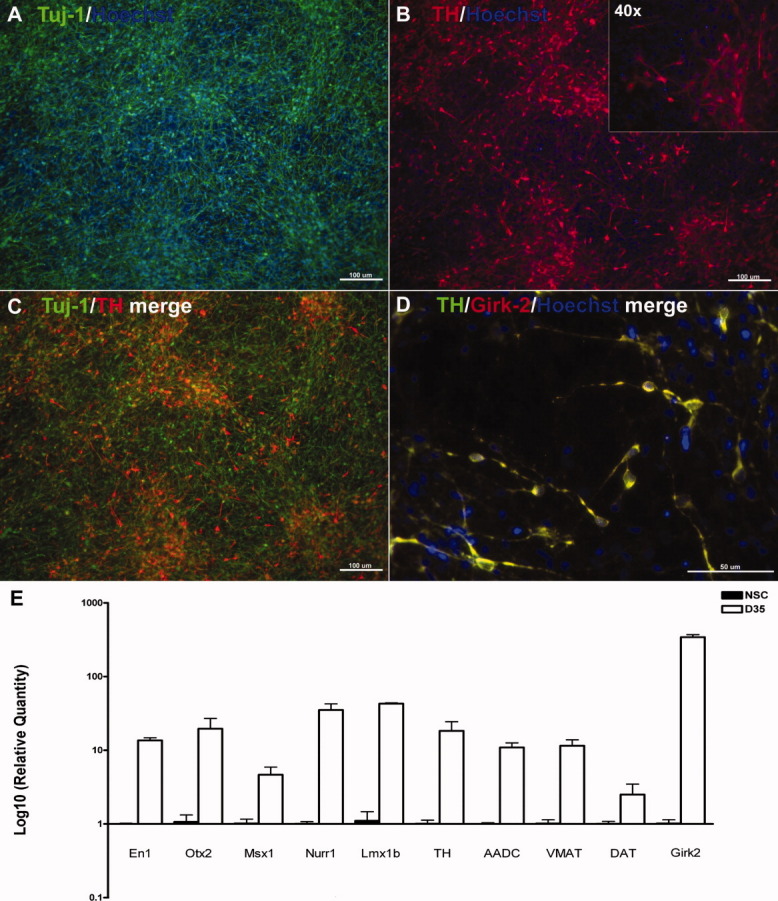
Efficient differentiation of induced pluripotent stem cell (iPSC)-derived NSCs into dopaminergic neurons. **(A–D):** iPSC-derived NSCs differentiated into midbrain dopaminergic neurons in defined media as seen by immunocytochemistry. The majority of the cells expressed β-III-tubulin and TH after 5 weeks of differentiation **(A–C)**. Coexpression of A9 marker Girk2 in TH^+^ dopaminergic neurons **(D)**. Differential expression of dopaminergic markers in dopaminergic neurons compared with NSCs by quantitative polymerase chain reaction **(E)**. All the examined markers were upregulated in dopaminergic populations compared with NSCs. Abbreviations: AADC, Aromatic L-Amino Acid Decarboxylase; DAT, dopamine transporter; NSC, neural stem cell; TH, tyrosine hydroxylase.

Treatment of iPSC-derived NSCs with Shh and FGF8 for 10 days followed by GDNF and BDNF for 3 weeks resulted in an efficient differentiation to dopaminergic neurons as a large numbers of cells expressed TH by immunocytochemistry (Fig. 2A–2C). For both iPSC lines, approximately 30% ± 5% of total cells were positive for TH, which was comprisable to the efficiency seen in hESC-derived NSC differentiation [5]. Importantly, nearly 100% of the TH-expressing neurons coexpressed nigral marker Girk2 (Fig. 2D), indicating that these neurons were of A9 dopaminergic neurons. Expression of additional midbrain and dopaminergic markers in iPSC-derived neurons was also assessed by real-time quantitative PCR. As expected, several markers including En1, Otx2, Lamx1b, Msx1, Nurr1, Lmx1b, Aromatic L-Amino Acid Decarboxylase (AADC), Vesicular Monoamine Transporter (VMAT), and dopamine transporter (DAT) were upregulated in dopaminergic populations compared with the expression in NSCs (Fig. 2E).

### iPSC-Derived Dopaminergic Neurons Survived and Ameliorate Behavioral Deficits in 6-OHDA PD Rats

**Figure 3:**
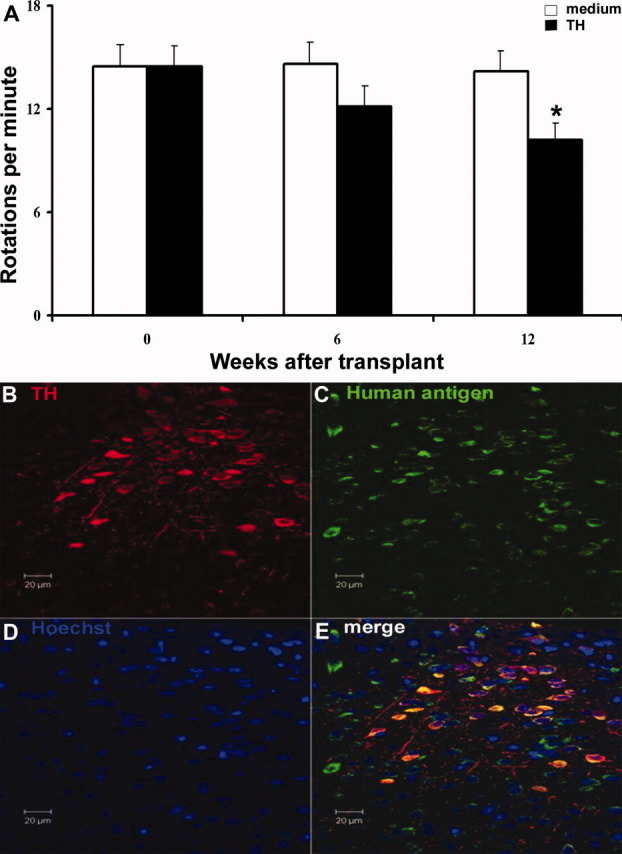
Transplantation of induced pluripotent stem cell (iPSC)-derived cells into 6-hydroxydopamine (6-OHDA) Parkinson's disease (PD) rats. iPSC-derived dopaminergic neurons engrafted and ameliorated behavioral deficits in a PD model. **(A):** Amphetamine-induced rotations in 6-OHDA rats grafted with iPSC-derived neurons (20 days after the neural stem cell stage) showed significant rotational improvement 12 weeks after transplantation. **(B–E):** Histological analysis of a representative brain section showed that donor cells (human antigen-immunopositive cells) coexpressing TH survived in the graft sites 12 weeks post-transplantation. Abbreviation: TH, tyrosine hydroxylase.

To investigate whether dopaminergic neurons derived from iPSCs exhibited functional properties, we transplanted the MR31-derived cells (20 days after the NSC stage) into 6-OHDA rats and conducted behavioral and histological studies, which we viewed as minimal criteria for assessing dopaminergic neuronal function. We first examined the behavior of sham-operated rats and rats received iPSC-derived dopaminergic neurons. As seen in Figure 3A, control rats that were transplanted with medium showed no attenuation of amphetamine-induced rotary behavior over the course of the experiment (12 weeks), whereas rats that were transplanted with grafts demonstrated significant rotational improvement at 12 weeks after transplantation (*p* < .05). Histological analysis revealed that donor cells (human antigen-immunopositive cells) coexpressing TH were found in all brains throughout the graft sites at 12 weeks (end of experiment) following the transplantation (Fig. 3B–3E). Cell counts in serial sections from one representative animal showed that the graft contained approximately 2,106 ± 313 TH-positive cells/mm^3^ human antigen-positive cells (donor-derived cells). This number of donor-derived dopaminergic neurons survived in the grafts was comparable with transplants with hESC-derived dopaminergic neurons [5]. We did not observe any structures resembling teratoma-like tissues by thorough histological analyses in the grafts in any animals regardless of receiving medium alone or iPSC-derive cells. Overall, these results were similar to what has been reported for hESC-derived cells transplanted in the same 6-OHDA rat PD model, indicating that iPSC-derived cells can survive in vivo and can ameliorate behavioral deficits in PD rats.

### Gene Expression Profiling of hESCs, iPSCs, and Cells Differentiated from Them

To further test the utility of iPSC-derived cells, we conducted a side by side whole genomic comparison of iPSCs and hESCs during dopaminergic differentiation using the defined medium protocol described earlier. Cells were harvested at three stages (ESC/iPSC, NSC, and dopaminergic neuron), and a transcriptome analysis was performed using Illumina arrays and pathways analysis software as described in “Materials and Methods” section and previously [18]. hESC data presented in this article is similar to previous analysis for the same population at a different passage number allowing for future comparisons of discrete data sets. The current analysis was limited to this data set (A total of nine samples: undifferentiated hESC line H9, iPSC lines MR31 and MMW2, NSCs derived from H9, MR31 and MMW2, and day 31 dopaminergic neurons derived from H9, MR31, and MMW2).

**Figure 4:**
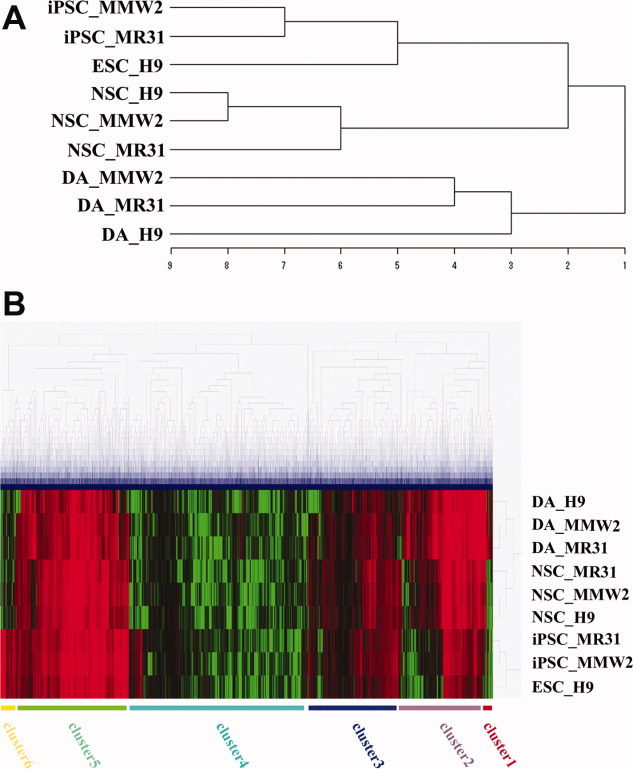
Whole genomic analysis of iPSCs at different stages of dopaminergic differentiation by Illumina bead microarray. A total of nine samples (undifferentiated human embryonic stem cell [hESC] line H9, iPSC lines MR31 and MMW2, NSCs derived from H9, MR31, and MMW2, and dopaminergic neurons derived from H9, MR31, and MMW2) were analyzed by Illumina array and the results demonstrated similarities between hESC and iPSCs. **(A):** Dendrogram of unsupervised one-way hierarchical clustering analysis of global gene expression data in hESCs/iPSCs, NSCs, and dopaminergic neurons derived from hESCs/iPSCs. **(B):** Unsupervised two-way hierarchical cluster analysis of differentially expressed genes illustrated in a heat map. The samples include three groups, hESCs/iPSCs, NSCs, and dopaminergic neurons. Expression values are presented as the log2 signal value of the given gene. Abbreviations: DA, dopaminergic; iPSC, induced pluripotent stem cell; NSC, neural stem cell.

Correlation clustering was performed to show that iPSCs were overall similar to hESCs rather than to differentiated cells, and NSC and dopaminergic neurons differentiated from them appeared more similar to each other. The overall correlation coefficient between each population was calculated by Pearson's rho (Table 1) and presented by a dendrogram analysis (Fig. 4A). This is consistent with our immunological and functional analysis, which suggests that iPSCs differentiate using similar cues and signals as hESCs, and that the overall process of differentiation and the end phenotype are similar to hESC-derived products.

**Table 1:** Correlation coefficients (*R*^2^) of paired samples on the microarray

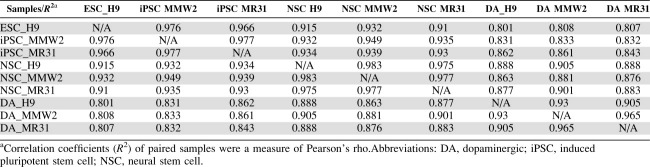

A closer analysis of the pluripotency network is shown in Table 2. As expected, many pluripotency genes including Oct4, Nanog, and Sox2 were highly expressed in iPSCs and hESCs. Of the genes shown, little difference was seen between the two iPSC lines and hESC line H9. The differences observed were similar in magnitude to those seen in comparisons among different hESC lines and the same line grown in different laboratories [18].

**Table 2:** Expression of genes representative of pluripotency or associated with NSC or dopaminergic differentiation in iPSCs/ESCs and their neural derivatives

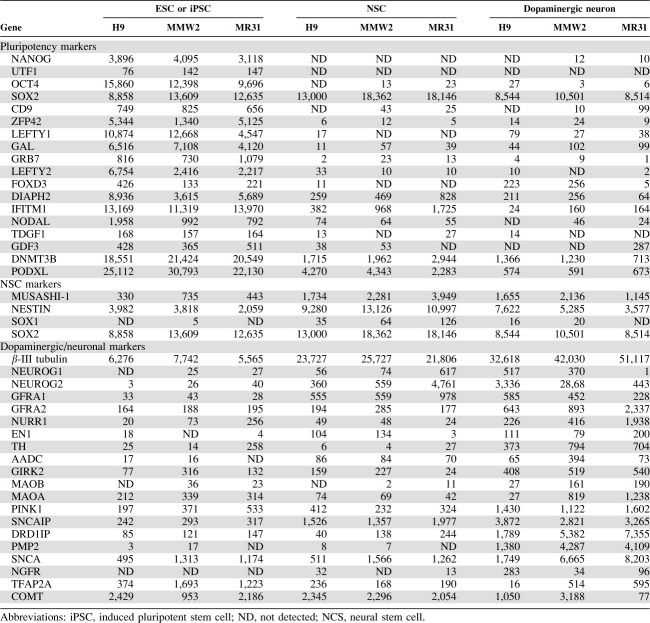

Given the different path to pluripotency and methods of differentiation, we looked at the expression of some genes/pathways that we and others have suggested might be different between hESCs and iPSCs such as genes involved in imprinting, cell cycle regulation, and reprogramming. Examination of imprinted genes which we thought may be different shows a small number of differences (Supporting Information Table 1). These included maternally expressed DLK1 and H19, and paternally expressed DLX5 and PEG3. It has been previously noted that the cell cycle in ESCs (pRb and p53 in particular) is regulated differently than in other somatic cell population [19]. Little difference at the transcriptome level was observed between the two iPSC lines and H9, suggesting that as cells transited to the iPSC state their cell cycle regulation changed appropriately. Likewise, the expression level of iPSC inducing genes (the three or four reprogramming factors) in the iPSC lines was comparable with H9 suggesting the exogenous induced genes had been silenced. Surprisingly, unlike other reports, we did not observe significant expression of fibroblast or mesodermal markers in undifferentiated iPSCs.

A more detailed analysis of the process of differentiation was performed by examining NSC gene expression comparisons and comparisons after cells had been differentiated to dopaminergic neurons. An unsupervised two-way hierarchical cluster analysis of differentially expressed genes across ESC/iPSC, NSCs, and dopaminergic neuron samples is also shown in Figure 4B. As expected, the heat map shows a clear separation of ESC/iPSC, NSC, and dopaminergic cell types, and a separation of six differentially expressed gene clusters in different colors. The hESCs and iPSCs types were strongly associated with cluster six (yellow), which was upregulated in hESCs and iPSCs, whereas downregulated in the other cell types. The red genes (cluster 1 and cluster 2) appear to be dopaminergic-specific, which were upregulated in the dopaminergic cells, whereas downregulated or expressed at low levels in undifferentiated and NSC samples. More specifically, Table 2 shows the expression levels of four markers representative of NSCs in iPSC- and hESC-derived NSC populations. No significant difference was observed among the lines. Of the 20 genes associated with dopaminergic/neuronal specification, differentiation, and maturation, or PD listed in Table 2, many of them including EN1, Nurr1, TH, AADC, and Girk2 were upregulated in dopaminergic populations derived from both the iPSCs and hESCs. Nevertheless, differences in expression of some genes among the lines were observed. These include SNCA, NGFR, MAOB, MAOA, TFAP2A, and COMT.

### Baculoviral Vector-Mediated Gene Transduction in iPSC-Derived NSCs and Neurons

**Figure 5:**
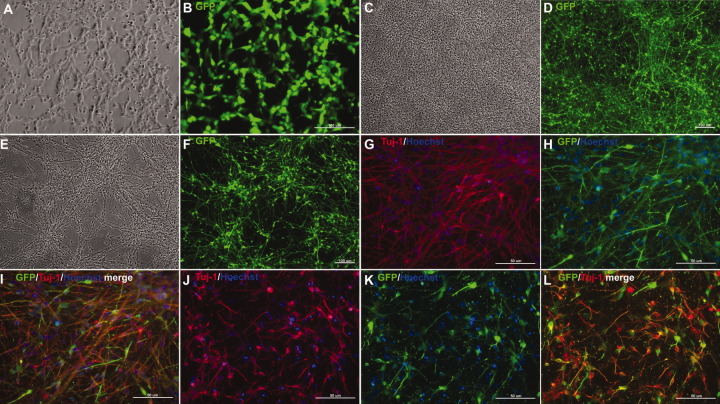
Efficient transduction of induced pluripotent stem cell (iPSC)-derived neural stem cells (NSCs) and neurons by baculoviral vector. iPSC-derived NSCs and neurons were transduced by a baculoviral vector carrying a GFP driven by the CMV promoter. **(A–L):** Fluorescence microscopy showed strong expression of GFP in iPSC-derived NSCs (87% of total cell were GFP^+^ by FACS) 24 hours post-transduction or day 36 neurons (90.3% of total cell were GFP^+^ by FACS) 2 days after transduction. Expression of GFP in human embryonic stem cell (hESC)-derived neurons was shown for comparison **(E, F)**. Immunostaining of β-III tubulin confirmed that the majority of neurons coexpressed GFP in both iPSC-derived neurons **(G–I)** and hESC-derived neurons **(J–L)**. Abbreviation: GFP, Green Fluorescent Protein; CMV, Cytomegalovirus; FACS, Fluorescence-Activated Cell Sorting.

One potential application of iPSCs is disease modeling. For the study of neurodegenerative disorders it is crucial to be able to introduce and express exogenous constructs in neuronal cells to assess gene function. Like other postmitotic cells, neurons present a particular challenge regarding the efficiency of gene transfer. To achieve efficient transduction in human neurons, we have developed a platform technology that utilizes an insect virus backbone (baculovirus) to deliver large payloads. Using this novel episomal baculoviral-based vector system (a nonintegrating gene delivery strategy), we tested this delivery system with a ubiquitous promoter (CMV) driving GFP in iPSC-derived NSCs and their differentiated neurons. As seen in Figure 5A and 5B, approximately 87% NSCs were expressing GFP 24 hours after transduction as assessed by Fluorescence-Activated Cell Sorting (FACS) analysis. Likewise, transduction is efficient in iPSC-derived neurons (36 days after NSC differentiation) as 90.3% of cells expressed GFP 2 days after transduction (Fig. 5C, 5D). Transduction in hESC-derived neurons was shown as comparison (Fig. 5E, 5F, 5J–5L). Immunostaining of β-III tubulin confirmed that the majority of neurons coexpressed GFP (Fig. 5G–5L). Importantly, expression of transgene in postmitotic neurons was maintained for at least 3 weeks, demonstrating the usefulness of this technology in genetic modification of human neurons including iPSC-derived neurons.

## DISCUSSION

A possible therapeutic approach to curing PD is to generate immune compatible dopaminergic neurons by reprogramming somatic cells (e.g., skin fibroblasts or blood cells) from PD patients and then differentiating them into dopaminergic neurons. To do this in a clinically compliant manner, it is important to show that scalable methods of GMP-compatible cell culture exist, and that iPSC lines are overall similar to hESCs so that the body of existing literature on hESCs can be utilized. It is equally important to show that iPSC lines can be obtained from disease models of PD, and that these lines can be propagated and differentiated appropriately, and used for comparative analysis. Finally, it is also important to show that a stage(s) where the effect of a mutation can be assessed and that the mutation can be corrected or a mechanism be identified.

In this article, we showed that iPSC lines derived from different somatic cell populations can be grown in a hESC-based protocol using a xenogenic-free defined medium and differentiation protocol optimized for hESCs. Using this 4-step scalable process (propagation of hESCs → generation of NSCs → induction of dopaminergic precursors → maturation of dopaminergic neurons), we tested two iPSC lines derived from different somatic cells (adult fibroblast and mesenchymal stem cell) and generated NSCs and subsequently dopaminergic neurons from them. We showed that each of the manufacturing steps could be performed using xeno-free defined conditions in iPSCs similar to hESCs. Neurons generated by this process appeared to be authentic A9 dopaminergic neurons as assessed by in vitro (marker expression) and in vivo (transplantation in PD animal model) assays. This observation was not entirely unexpected as it has been observed that the reprogramming factors are not needed forever. Indeed, once the cells are reprogrammed, they express endogenous pluripotency genes and silence the exogenous ones. Thus, like ESCs or other pluripotent cells, iPSCs should readily differentiate into appropriate lineages and respond in a manner indistinguishable from ESCs. Indeed, this observation has been utilized cleverly by several groups to develop zero-footprint technology [20,21] that allows one to reprogram somatic cells using factors or genes that can then be permanently eliminated leaving cells that theoretically, at least, should be indistinguishable from ESCs derived in a conventional fashion, and thus media and reagents developed for ESCs should work with iPSCs.

Our results of directly comparing hESCs with iPSCs suggest that it is true that iPSCs are similar to hESCs. Both functional studies and transcriptome analyses suggest that overall iPSCs derived from somatic cells are largely similar to hESCs. We focused our transcriptome analysis in particular on selected pathways that we and others have suggested may be different between ESCs and iPSCs given the different histories of the ancestor cell that generates a pluripotent population. These include maternal and paternal allele-specific gene expression (imprinting), cell cycle, and senescence, and subtle biases in differentiation due to the source of cells and incomplete reprogramming. Although we did not detect any significant differences, we emphasize that subtle biases are probably best tested in competitive functional assays. Our transplant results suggest that this may be a useful model in which mixed labeled populations can be transplanted in a competitive assay akin to that descried earlier by Jaenisch and coworkers where they showed mouse iPSCs differentiated into functional neurons and glia after transplantation into the developing brain [10].

Given the overall similarity and the ability to bypass the immune response, interest in using iPSCs for therapy has been high. Recently, iPSC lines from sporadic PD patients have been generated [8] and several groups have now shown that gene targeting can be performed with roughly the same efficiency as in hESCs, and that iPSCs from people carrying known mutations can be harvested and propagated [22,23]. Although PD is usually sporadic, genetic studies have, however, identified mutations in several genes, including *LRRK2, parkin, α-synuclein, uchL1, PINK1, DJ-1*, and *ATP13A2*, in familial PD. We, therefore, initiated an effort to generate iPSC lines from familial PD patients with defined mutations. As a proof-of-principle experiment, we derived an iPSC line from skin biopsies obtained from a PD patient with a defined mutation in *LRRK2* as mutations in *LRRK2* account for approximately 7% of familial PD cases and a significant portion of sporadic PD cases [24,25]. Using the identical differentiation protocol, we derived Sox1^+^/Nestin^+^ NSCs from the *LRRK2* iPSC line. Further analysis of potential phenotype during dopaminergic differentiation associated with the mutation in familial PD patient lines is the scope of our future work.

Full utilization of iPSCs for personalized medicine and disease modeling requires the development of methods of efficient genetic modification including gene targeting and efficient expression or reporters. Recently, it has been shown that gene targeting in iPSCs can be achieved by homologous recombination mediated by zinc finger nucleases [22,23]. Efficient gene transfer in iPSC-derived differentiated cells such as neurons would be valuable for both therapy and gene discover, but has been a challenge because of the difficulty of transfecting postmitotic cells. Nonviral physical- or chemical-based methods such as electroporation, nucleofection, and lipofection are inefficient in transfecting postmitotic cells [26]. Viral vectors such as lentiviral and retroviral systems can transduce neurons with high efficiency, but are primarily used for stable genomic modification and transduction is associated with random integration of transgene. Here, we used an episomal baculoviral-based vector to achieve efficient expression of transgene in iPSC-derived NSCs and particularly in postmitotic neurons. This nonintegration method of high efficiency transfection in human neurons may enhance the utilization of iPSCs for disease modeling as it provides a powerful tool to study neuronal cell biology.

In conclusion, we believe that we have provided strong evidence of the utility of the iPSCs approach to both gene discovery and therapy.

## DISCLOSURE OF POTENTIAL CONFLICTS OF INTEREST

The authors indicate no potential conflicts of interest.
