# Supplementary figures and images for: Efficient Generation of Functional Dopaminergic Neurons from Human Induced Pluripotent Stem Cells Under Defined Conditions
Source: Stem Cells. 2010 Aug 16;28(10):1893–904. doi: 10.1002/stem.499 (PMC2996088; doi:10.1002/stem.499)

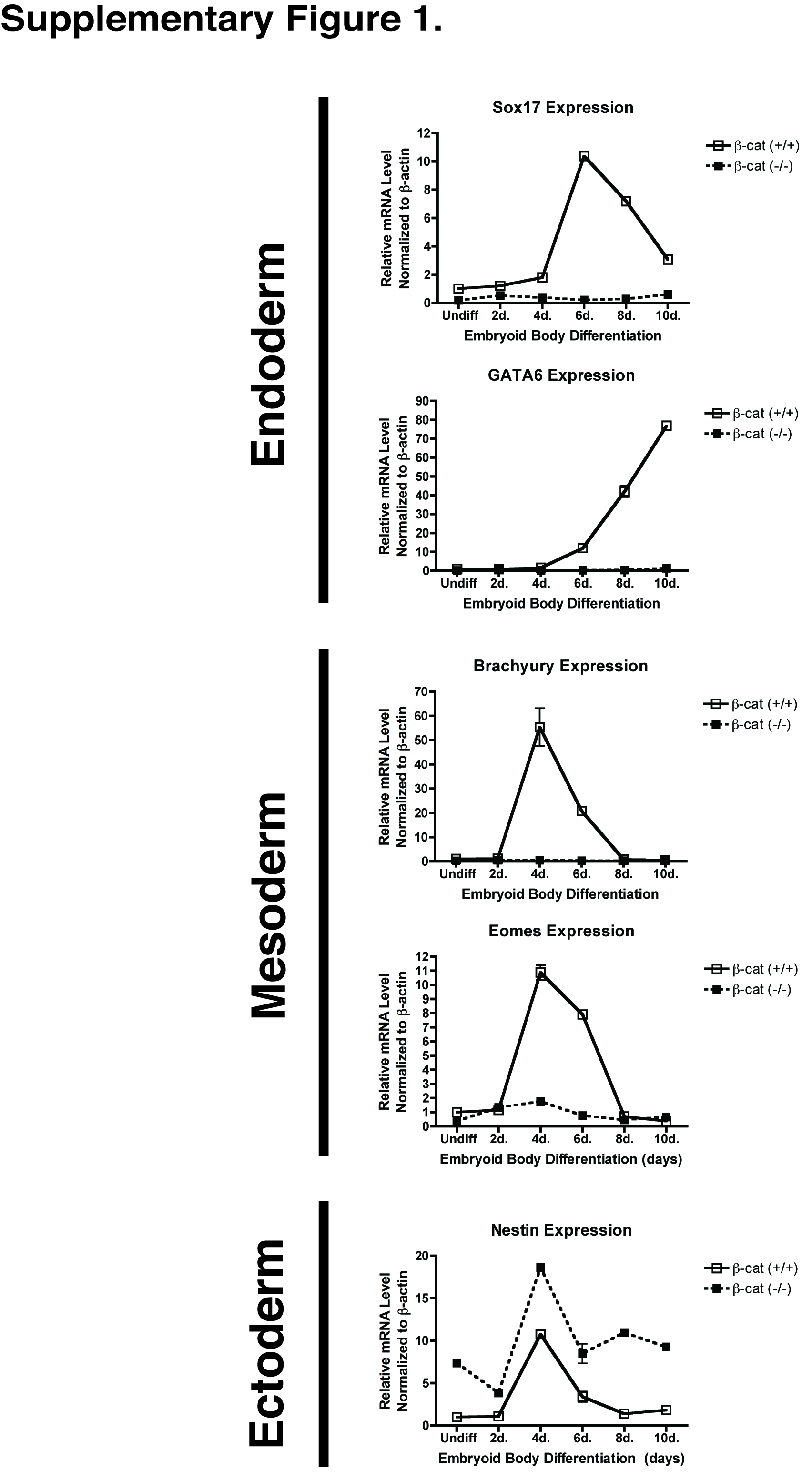

Supplement: Supplementary file 1 [file stem0028-1893-SD1.tif]

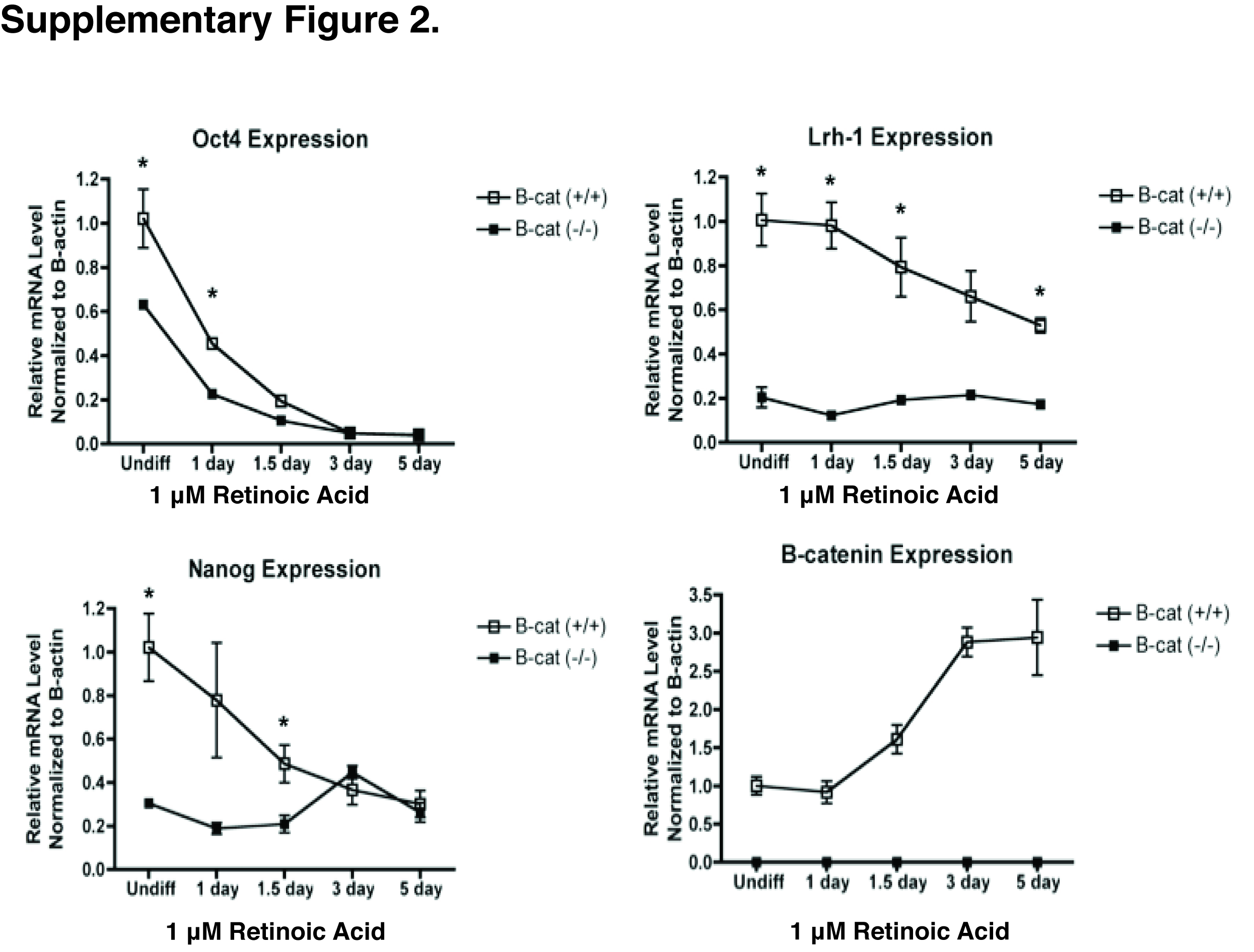

Supplement: Supplementary file 2 [file stem0028-1893-SD2.tif]

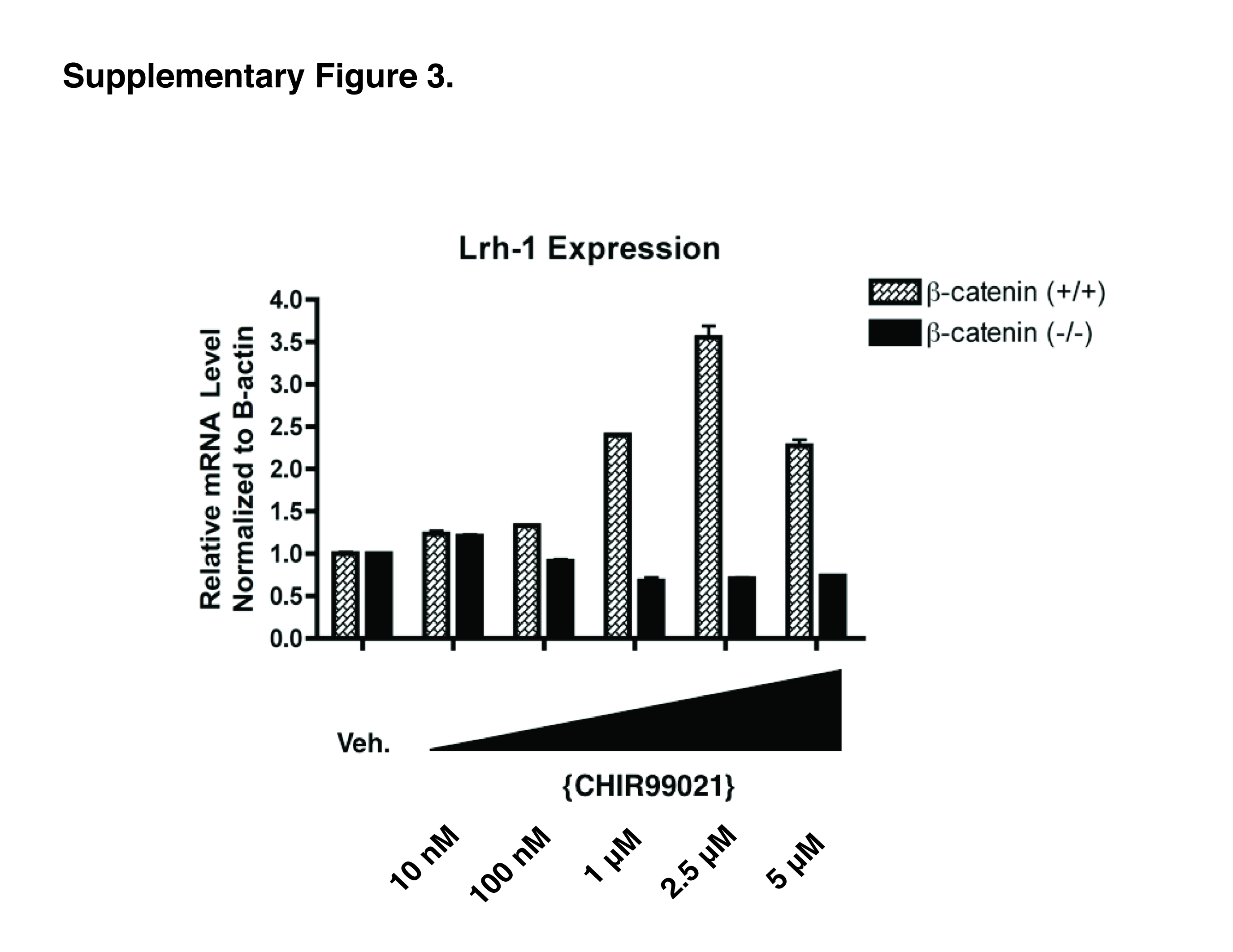

Supplement: Supplementary file 3 [file stem0028-1893-SD3.tif]

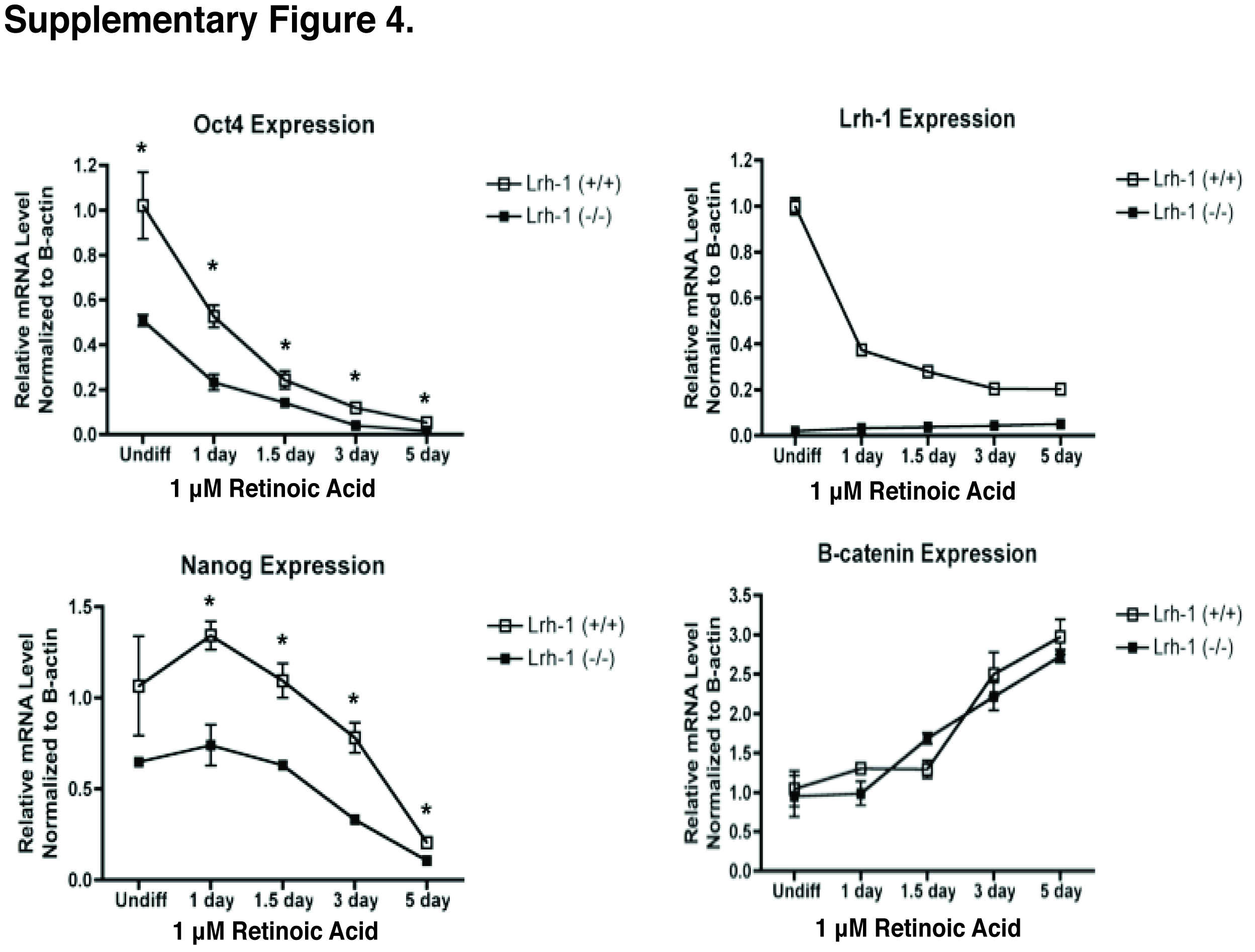

Supplement: Supplementary file 4 [file stem0028-1893-SD4.tif]

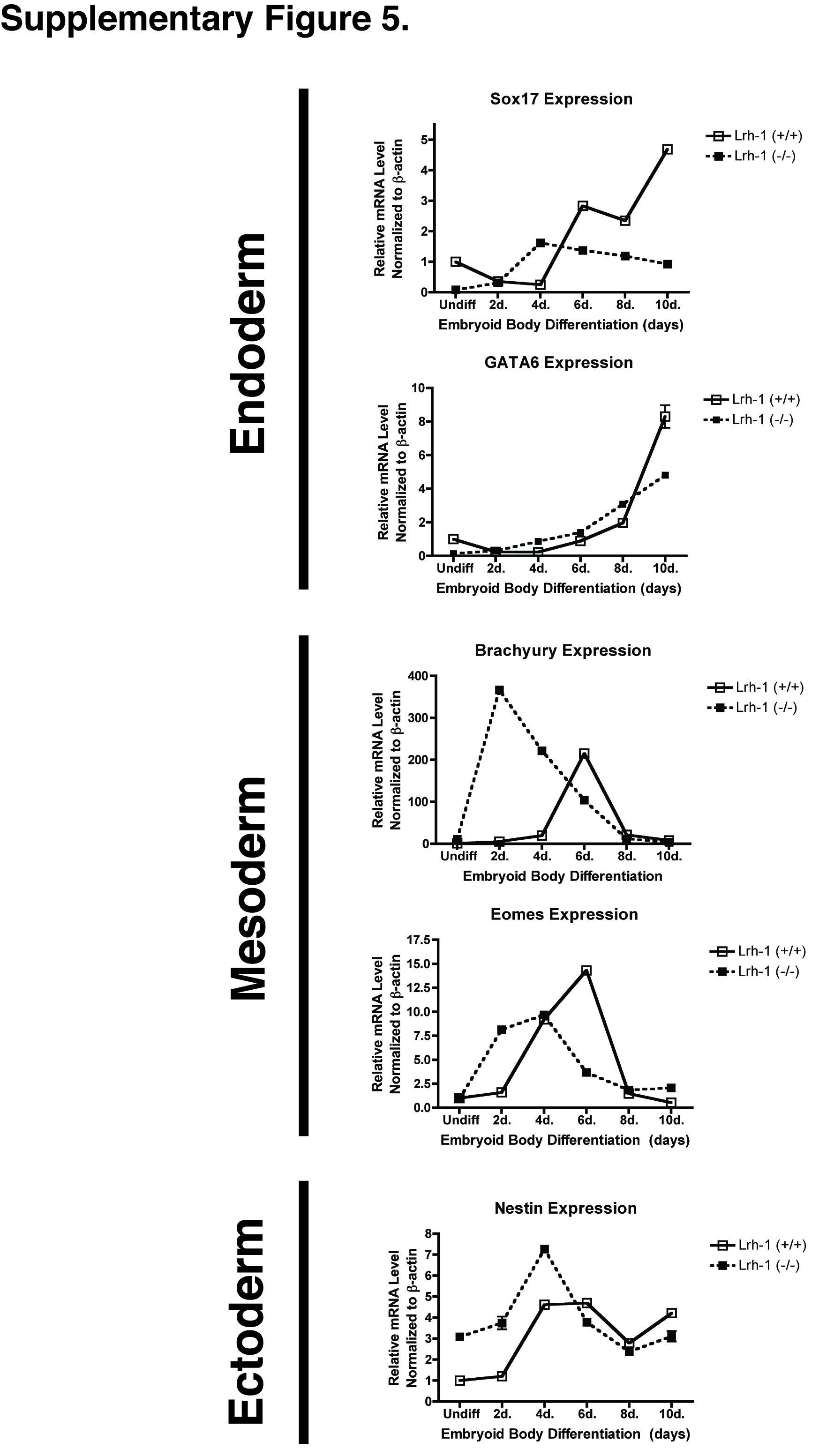

Supplement: Supplementary file 5 [file stem0028-1893-SD5.tif]

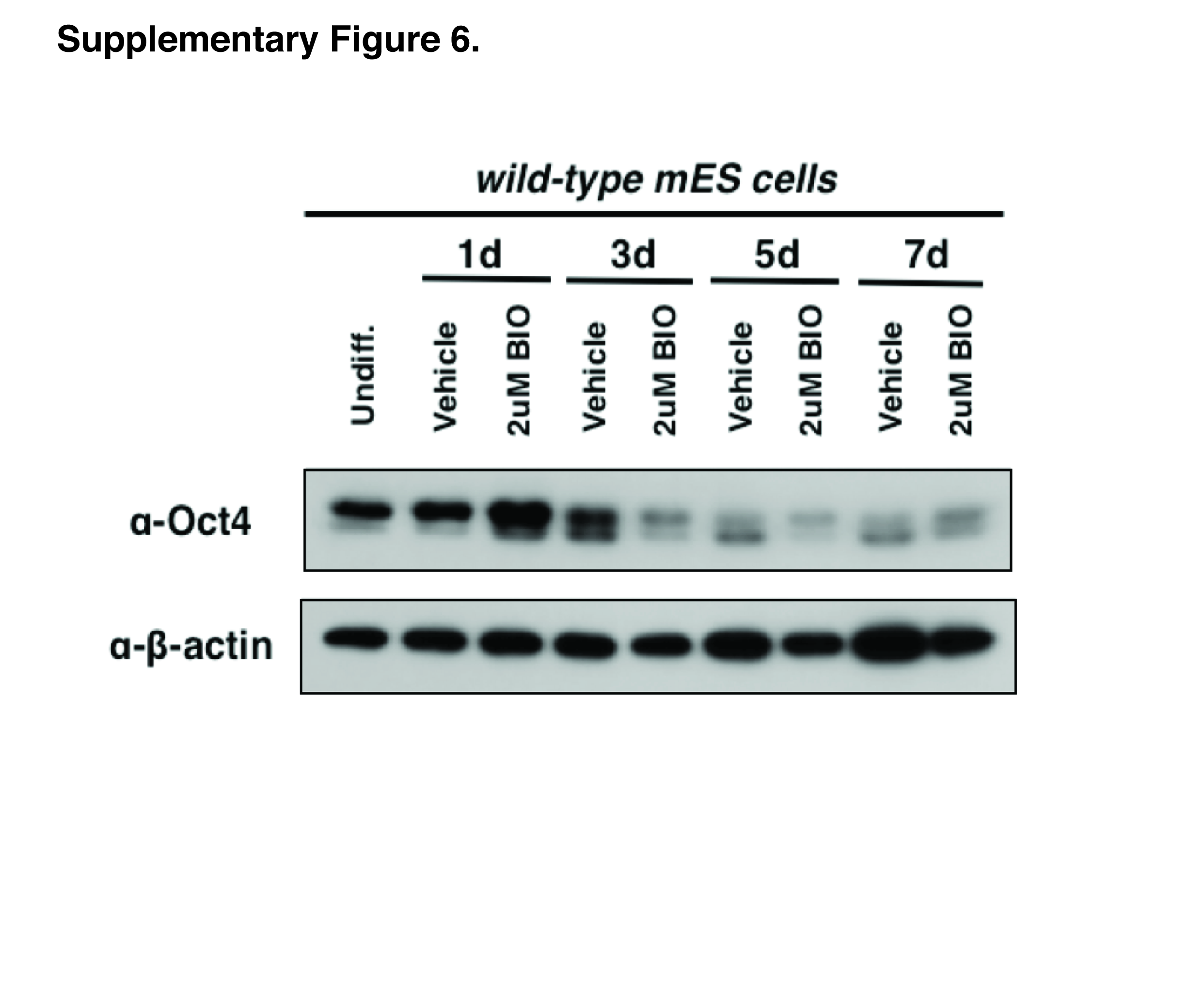

Supplement: Supplementary file 6 [file stem0028-1893-SD6.tif]

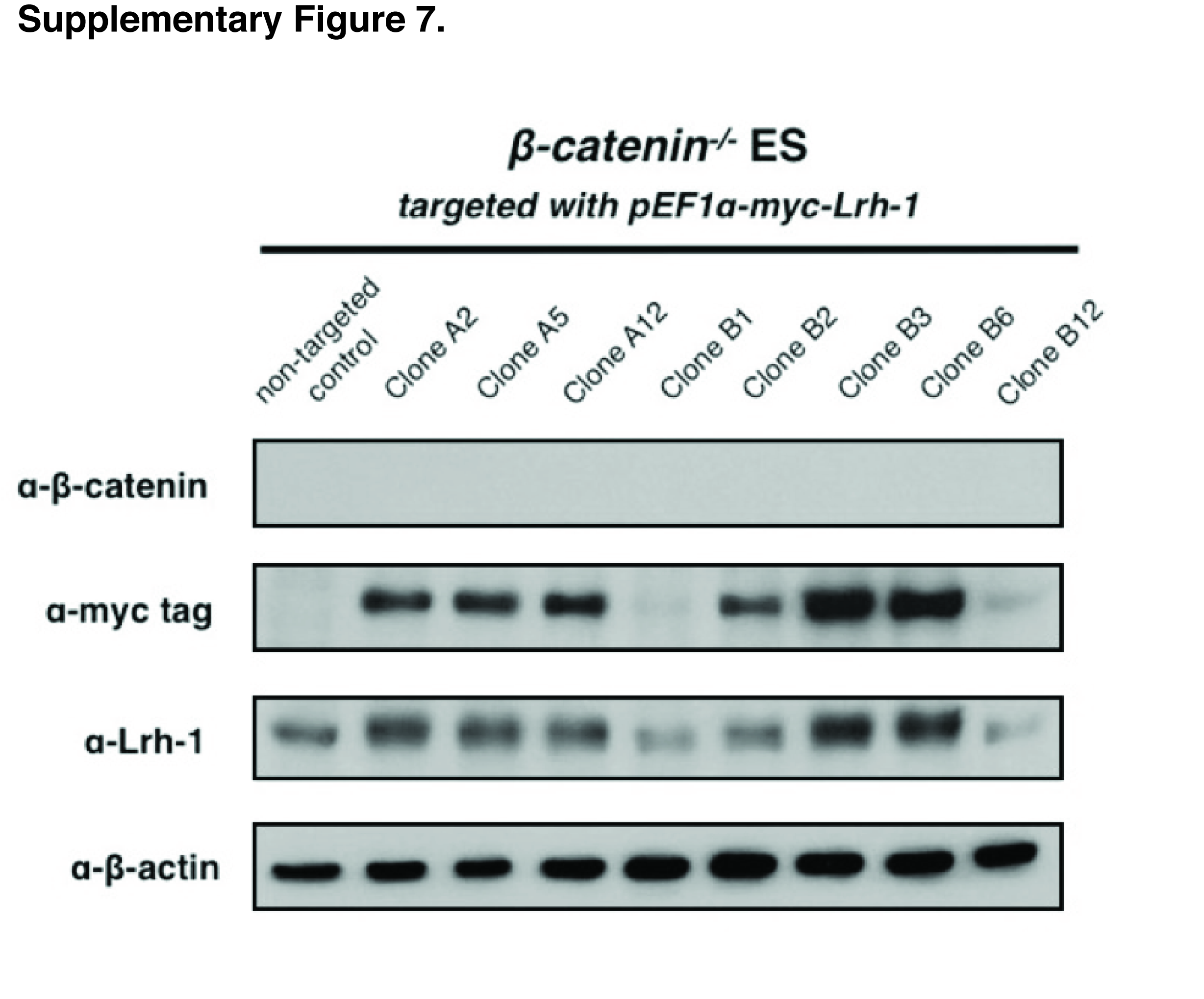

Supplement: Supplementary file 7 [file stem0028-1893-SD7.tif]
