# Supplementary material for: Efficient Generation of Functional Dopaminergic Neurons from Human Induced Pluripotent Stem Cells Under Defined Conditions
Source: Stem Cells. 2010 Aug 16;28(10):1893–904. doi: 10.1002/stem.499 (PMC2996088; doi:10.1002/stem.499)
Supplement: Supplementary file 8 [file stem0028-1893-SD8.doc]

**Supplementary Table 1: Antibodies and Conditions**

| **Target** | **Company** | **Clone** | **Western Blot** | **Chromatin-IP** |
| --- | --- | --- | --- | --- |
| **Lrh-1** (NM_030676) | R&D Systems | H2325 | (1/2000) | 1ug ab/  300 ug lysate |
| **β-catenin** (NM_007614) | Abcam | Ab2982 | (1/2500) | 1ug ab/  150 ug lysate |
| **Oct4** (NM_013633) | Santa Cruz  Biotechnologies | H134 | (1/1000) |  |
| **Nanog** (NM_028016) | Millipore | Ab5731 | (1/2000) |  |
| **TCF-3** (NM_009332) | Merrill Lab  (Pereira et al.) |  |  |  |
| **Myc-tag** | Clontech | 631206 | (1/1000) |  |
| **β-actin** (NM_007393) | Sigma | 3G4-F9 | (1/5000) |  |
| **Tbx3** (NM_011535) | Santa Cruz  Biotechnologies | A20 | (1/1000) |  |
| **Klf4** (NM_010637) | Millipore | Ab4138 | (1/500) |  |

Supplementary Table 2: qRT-PCR Primers

| **Target** | **Forward Primer** | **Reverse Primer** |
| --- | --- | --- |
| **mLrh-1** (NM_030676) | GAAGCTGGAAGCCGTAAGAG | GGCTTTCTTCTGCTGCTTCAA |
| **mβ-catenin** (NM_007614) | GTGCAATTCCTGAGCTGACAA | ATGATGGCATGTCTGGAAGCT |
| **mOct4** (NM_013633) | GTTGGAGAAGGTGGAACCAA | CCAAGGTGATCCTCTTCTGC |
| **mNanog** (NM_028016) | ATGCCTGCAGTTTTTCATCC | GAGGCAGGTCTTCAGAGGAA |
| **mβ-actin** (NM_007393) | AACCCTAAGGCCAACCGTGAA | ACAGCCTGGATGGCTACGTA |
| **mNestin** (NM_016701) | CTTGCAGACACCTGGAAGAA | TGAGGACAGGGAGCACAGAT |
| **mGata6** (NM_010258) | CACTACCTTATGGCGTAGAA | TCCTGGTTTGAATTCCTTCT |
| **mSox17** (NM_011441) | GATGAACGCCTTTATGGT | CAAGACTTGCCTAGCATCT |
| **mAFP** (NM_007423) | CTCTGGAGGATAAATTCATCT | TGCTTCACCAGGTTAATGA |
| **mBrachyury** (NM_009309) | TCTCCAACCTATGCGGACAAT | TAGGTGGGCTGGCGTTATGA |
| **mEomes** (NM_010136) | TCACCAATAACAAAGGTGCAA | TGAGTCTTGGAAGGTTCATTCA |

Supplementary Table 3: Chromatin-IP Primers

| **Target** | **Forward Primer** | **Reverse Primer** |
| --- | --- | --- |
| **mLrh-1 Ts(FL)-1** | GCCCAATGACCAATATACAAGCAAGC | GAAGGACAGACTCTGCTCTTCTGAC |
| **mLrh-1 Ts(FL)-2** | GTATTTGCAGAGGTCAGAAGAGCAG | GGGTTCTTCTGCAGCCCAGAGTGT |
| **mLrh-1 Ts(ES)-1** | TCTCACGGAAGCGGAGGATGT | GAACAGCCCGGTTTCTAAAGAC |
| **mLrh-1 Ts(ES)-2** | TAACCAGGGAAGATCACTGGCT | ACTCAGTTGAGCCAATAATGCG |
| **Axin-2** | GATCACTGGCTCCCCGAG | TACAGCAAAGCTCTCCTTTGG |
| **mLrh1 (intronic)** | TAGGTGAGCATATTCTAACA | ATCGGAGCTGGATCAAAC |
| **Oct4 PP** | ATAGCGCTCGCCTCAGTTTCT | TGGAAAGACGGCTCACCTAG |
| **Oct4 PE** | AAGTTGTCCCCAGGGGAGCCAT | TATCTGACTTCAGGTTCAAAG |
| **Oct4 (intronic)** | GGAGGGAGAACTGAGAATCTTGAGG | ATTGCACCTCCAGTCCTCCAGAGTT |
| **Nanog -4.5kb** | CTGTACCAAACCTTTGTAGAA C | CAAAATGCATGCGAGTCCTCTA |
| **Nanog 3’UTR-A** | CATAGCTCTAATTGTCTTTGA | ACCTTTGGTCCCAGCATTCA |
| **Nanog 3’UTR-B** | TACTCTCGAGGATGAGACAGAA | TATCGAGATGCTGTCTTACTAT |
| **Tbx3 -12kb** | TCAGCATGCGAATCCCTAGCA | TGCTTGGTTGATGACTGGGTAT |
| **Klf4 -4.2kb** | GCATAAAGCAGTCTATT | TTCTTTTTGAGGCAGGGGTGTTA |
| **Klf4 -3.2kb** | CTGCATGTCATTCCTGGTGAT | CTGTGTGGTTTTGCTGTGTTT |
| **intronic** | TAAGTTGTTACTTTAATGACT | ATGGTATGATTCTTACTTGA |
